# Vitamin A Metabolism and Adipose Tissue Biology

**DOI:** 10.3390/nu3010027

**Published:** 2011-01-06

**Authors:** Simone K. Frey, Silke Vogel

**Affiliations:** Department of Medicine and the Institute of Human Nutrition, Columbia University, 630 W 168th Street, P&S 1512, New York, NY 10032, USA; Email: mail@simonefrey.de

**Keywords:** retinol, retinoic acid, adipose tissue, cellular retinol-binding protein, beta-carotene, adipogenesis

## Abstract

In recent years, the importance of vitamin A in adipose tissue biology, obesity and type II diabetes has become apparent. This review focuses on recent developments within the area of vitamin A and adipose tissue biology. Adipose tissue has an active vitamin A metabolism as it not only stores vitamin A but retinol is also converted to its active metabolite retinoic acid. Several mouse models point to a relationship between vitamin A metabolism and the development of adiposity. Similarly, *in vitro* studies provide new molecular mechanisms for the function of different forms of vitamin A and retinol- or retinoic acid-binding proteins in adipose tissue.

## 1. Introduction

Vitamin A must be obtained from the diet by intake of either food containing preformed vitamin A (e.g., red meats) or provitamin A carotenoids (e.g., carrots, green leafy vegetables). Most actions of retinol are mediated by its metabolite retinoic acid, which is synthesized intracelluarly in target tissue from retinol [1]. In the first step, retinol is reversibly oxidized to retinaldehyde. Two major enzyme classes, cytosolic aldehyde dehydrogenases (ADHs) and microsomal short-chain dehydrogenases (SDR) have been proposed to catalyze this reversible reaction [2,3]. In the second step, retinaldehyde is irreversibly oxidized to retinoic acid. The enzyme class of cytosolic retinaldehyde dehydrogenases (RALDH) has been clearly identified to catalyze this step [4]. 

Retinoic acid exerts its pleiotropic effects mostly by controlling the expression of over 500 genes through binding to and activating retinoic acid receptors (RAR) and retinoid X receptors (RXR), both of which are members of the nuclear hormone receptor family [5,6]. In addition to its oxidation to retinoic acid, retinol can be esterified and stored as retinyl ester in hepatic stellate cells or, to a smaller extent, in extrahepatic tissues including adipose tissue (see below). 

The liver plays a central role in vitamin A physiology. Postprandially, vitamin A is delivered to the liver as a constituent of chylomicron remnants. In the liver, retinol is re-esterified to retinyl ester by lecithin:retinol acyltransfrase (LRAT) and stored in hepatic stellate cells, the major storage site for vitamin A in the body [7]. Retinol bound to retinol-binding protein (RBP; RBP4) is secreted from the liver to maintain serum vitamin A levels and to deliver retinol to extrahepatic target tissues for intracellular retinoic acid synthesis [8]. RBP contains one binding site for retinol and in plasma RBP circulates as a 1:1 complex with transthyretin. Plasma RBP-retinol levels are under homeostatic regulation and are maintained at levels between 2 and 3 μM [8]. 

Vitamin A, its metabolites, and its synthetic analogs are collectively known as retinoids. The importance and essentiality of retinoids to human health, facilitating normal vision, reproduction, immune function, and cell differentiation have been widely shown and established [9]. In recent years, the importance of retinoids in adipose tissue biology, obesity and type II diabetes has become apparent. This review will focus on recent new developments within the area of retinoids and adipose tissue biology. 

## 2. Adipose Tissue

### 2.1. General

White adipose tissue is the major tissue for storage of excessive energy in the form of triglycerides, releasing fatty acids into the circulation in times of fasting and starvation. In addition to being an energy reserve for fatty acids, adipose tissue is also an active endocrine organ secreting many different substances, including adipokines with paracrine or endocrine function [10,11,12]. It has become increasingly clear that adipose tissue is an essential regulator of whole-body energy homeostasis [13]. In light of increased rates of obesity in countries worldwide, and the consequent increase in associated morbidity, understanding adipose tissue development and physiology has become of increasing importance. Below we highlight the role of retinoid metabolism in this context.

### 2.2. Retinoid Metabolism in Adipose Tissue

Retinoids in adipose tissue are derived from several sources. Retinol bound to retinol-binding protein in the circulation is one source used by adipose tissue. Furthermore, retinyl ester and β-carotene present in chylomicrons postprandially reach adipose tissue. Lipoprotein lipase present on endothelial cells facilitates the uptake of these postprandial retinoids into adipose tissue [14]. The presence of β-carotene monoxygenase type 1 (Bcmo1) in adipose tissue enables the conversion of β-carotene to retinaldehyde [15,16].

It has been estimated that adipose tissue stores 10–20% of total retinoids in the body as retinyl esters [17]. Under conditions of dietary vitamin A deficiency, the adipose retinol/retinyl ester stores are readily mobilized as evidenced by decreasing levels [18,19,20]. 

Mice deficient in lecithin:retinol acyltransfrase (LRAT), the enzyme that catalyzes the esterification of retinol, have very low hepatic retinyl ester stores, but adipose retinyl ester levels in these mice is higher than in wild-type mice. This indicates that adipose tissue can store retinol as retinyl esters when hepatic retinol storage is defective. These data also indicate that LRAT is the dominant enzyme for hepatic retinol esterification but in adipose tissue a different enzyme, and a yet unidentified acyl-CoA:retinol transferase (ARAT), may be important for retinol esterification [21,22]. Diacylglycerol acyltransferase type 1 (DGAT1) has been proposed to be this ARAT [23,24] however, in mice lacking both DGAT1 and LRAT, adipose retinyl ester levels were not significantly different from those in control mice [25], suggesting the possible existence of an additional enzyme involved in esterifying retinol. 

In addition to esterification, retinol is converted to retinaldehyde and retinoic acid in adipose tissue. Both, aldehyde dehydrogenase (ADH1), catalyzing the first step in retinol oxidation and retinaldehyde dehydrogenase (Raldh1), responsible for retinoic acid synthesis, have been identified in adipose tissue [26]. The role of the latter as it may relate to adipocyte differentiation is discussed below [26]. 

Intracellularly, protein binding may be an important mechanism to direct retinol towards enzymes that metabolize retinol to retinaldehyde and then to retinoic acid or retinyl esters; this would protect retinol from nonspecific oxidation/isomerization [27,28,29]. These roles have been attributed to intracellular proteins that specifically bind retinol; they are known as cellular retinol-binding proteins (CRBP) [27,29,30]. Thus far, three types, CRBP-I, CRBP-II and CRBP-III, have been described in murine tissues [27,31,32]. Together with cellular retinoic acid-binding proteins (CRABP) and fatty acid-binding proteins (FABP), they belong to the family of intracellular lipid-binding proteins (iLBP) [30]. It is currently thought that the primary role of CRBP-I and CRBP-III is to facilitate the esterification of retinol to retinyl ester catalyzed by lecithin:retinol acyltransferase (LRAT) [31,33]. The lack of CRBP-I in a mouse model leads to a 50% reduction of vitamin A storage in the liver and higher hepatic turnover of vitamin A in mice [33,34]. Similarly, we have shown that the absence of CRBP-III leads to decreased retinol esterification during lactation resulting in decreased milk retinyl ester levels in mice [31].

CRBP-I and CRBP-III have high protein homology but they differ in their affinity for retinol and in their tissue and cellular expression patterns [31,32,35]. CRBP-I binding affinity for retinol is almost two orders of magnitude greater than that of CRBP-III. Both binding proteins are expressed in adipose tissue, with CRBP-I expression restricted to preadipocytes and CRBP-III expression localized to endothelial cells and mature adipocytes [31,32,35]. This localization suggests that they may have different functions and this possibility is discussed below in more detail. 

In addition to CRBP, other members of the intracellular retinoid-binding protein family, cellular retinoic acid-binding proteins (CRABP), which bind retinoic acid with high affinity, are also expressed in adipose tissue and are involved in delivering RA into the nucleus. Of particular interest is CRABP-II as it has recently been shown to be involved in controlling adipogenesis (see below). 

## 3. Results from Dietary Interventions in Rodents and Knockout Mice

### 3.1. Retinoid Supplementation and Body Weight

While the association between dietary vitamin A intake and obesity in humans is difficult to assess, several studies employing rodent models have examined this relationship with various outcomes [36,37,38,39,40,41]. Supplementation of obese rats with high levels of dietary vitamin A (129 mg of vitamin A/kg) led to significant weight loss compared to a control group receiving lower levels of dietary vitamin A (2.6 mg of vitamin A/kg) [36]. A similar supplementation regimen in lean mice had no effect [37]. In contrast, short-term supplementation with retinoic acid resulted in weight loss, decreased adiposity and increased insulin sensitivity in several lean and/or obese mouse models [37,38,39,40,41]. Similarly, a diet deficient in vitamin A (no detectable vitamin A levels) led to an increase in adiposity in mice [40].

### 3.2. Several Knock-Out Models Allow Insights in Retinoid Metabolism in Adipose Tissue

#### 3.2.1. Retinol Dehydrogenase Type 1 (RDH1)

Apart from dietary studies, studies of genetically engineered mice provide further insights into the role of retinoid metabolism in adipose tissue biology and energy metabolism. Several mouse models underscore the role of intracellular retinaldehyde and/or retinoic acid synthesis in the development of adiposity. Napoli and coworkers have shown that retinol dehydrogenase (RDH1), which catalyzes the first reversible oxidation of retinol, is important during conditions of marginal intake of vitamin A [42]. In mouse models lacking RDH1, both retinol and retinyl ester levels were significantly increased during low dietary vitamin A intake (<4 IU/g) but not under conditions of excess vitamin A intake (>15 IU/g) [42]. Intracellular retinoic acid levels remained unchanged. Interestingly, a significant increase in body weight on a vitamin A restricted diet occurred in RDH1 knockout mice compared to wild-type mice [43]. The weight gain was mostly due to increased fat mass, but molecular mechanisms remain to be determined, especially as RDH1 is predominantly expressed in the liver but not adipose tissue. 

#### 3.2.2. Retinaldehyde Dehydrogenase Type I (Raldh1)

Further evidence that intracellular retinoid metabolism affects both adiposity and glucose homeostasis comes from a mouse model deficient in retinaldehyde dehydrogenase, type 1 (Raldh1), an enzyme responsible for the oxidation of retinaldehyde to retinoic acid. The absence of Raldh1 led to a significant increase in retinol and retinaldehyde levels in adipose tissue. Metabolically Raldh1 knockout mice had altered adipogenesis and glucose homeostasis. Specifically, when fed a high-fat diet, Raldh1 knockout mice remained leaner, and more insulin sensitive and glucose tolerant, than control mice [26]. 

#### 3.2.3. Beta-Carotene Monoxygenase (Bcmo1)

Like preformed retinol, β-carotene is a precursor for the synthesis of retinaldehyde and retinoic acid [44,45]. β-carotene presents a precursor as it can be enzymatically cleaved resulting into the formation of one or two retinaldehyde molecules. The latter can subsequently be reduced to obtain retinol.

The enzyme β-carotene monoxygenase type I (Bcmo1), present in intestine, testes and adipose tissue, can convert β-carotene into retinaldehyde via central cleavage of β-carotene. The lack of Bcmo1 in a murine model affected vitamin A homeostasis as well as lipid and adipose tissue metabolism. Mice deficient in Bcmo1 developed dyslipidemia and hepatic steatosis [15]. In mice with diet-induced obesity, the lack of Bcmo1 was associated with a significant increase in adiposity, elevated serum free fatty acid levels and fatty liver compared to wild-type mice [15].

#### 3.2.4. Cellular Retinol-Binding Protein Types I and III (CRBP-I&III)

As described above, both CRBP-I and CRBP-III are expressed in adipose tissue but in different cellular compartments [32,46,47]. We have recently reported that both CRBP-I and CRBP-III play important roles in adipose tissue biology and energy homeostasis [46,47]. The absence of CRBP-III did not result in changes of intracellular retinoid levels in adipose tissue. However, when fed a high-fat diet, mice lacking CRBP-III showed significantly decreased adiposity and developed less hepatic steatosis, as evidenced by decreased liver triglyceride levels, as compared to control mice [47]. Lower serum fatty acid levels in CRBP-III knockout mice may offer an explanation for lower hepatic TG accumulation [47]. 

This is contrasted by results from mice lacking CRBP-I. A high-fat diet resulted in a significant increase in adiposity in CRBP-I knockout mice compared to wild-type mice [46]. However, CRBP-knockout mice remained insulin sensitive and glucose tolerant despite diet-induced obesity. The positive impact of adipose tissue expansion in the absence of CRBP-I mice may be explained by increased PPARγ expression, an increased frequency of small adipocytes, and higher serum adiponectin levels compared to control mice [46].

#### 3.2.5. Retinol-Binding Protein (RBP/RBP4)

In addition to the role of intracellular retinoid metabolism in energy homeostasis, it has also been suggested that retinol-binding protein (RBP/RBP4) synthesized and secreted from adipose tissue—not hepatic RBP4—may act as an adipokine that negatively affects blood glucose homeostasis in mice [17,48]. Accordingly, mice deficient in RBP/RBP4 remained more insulin sensitive under conditions of diet-induced obesity [48]. Yang and Graham [48] showed a strong positive relation between increased serum RBP4 levels and the incidence of type II diabetes and insulin resistance. Their results were based on findings in mice lacking the glucose transporter 4 (GLUT4) specifically in adipose tissue (GLUT4 KO) rendering the mice insulin resistant. Interestingly, the lack of adipose tissue GLUT4 was associated with increased expression of RBP4 in adipose tissue but not in liver resulting in increased serum RBP4 levels in the GLUT4 KO mouse model. Consistent with these data, mice deficient in RBP4 remained more insulin sensitive under conditions of diet-induced obesity [48]. Therefore it has been speculated that adipocyte derived RBP4—not hepatic RBP4—may be an adipokine and contribute to the development insulin resistance [48]. The molecular mechanisms are unclear. Chronic RBP4 elevation may lead to increased hepatic glucose production through stimuating the hepatic enzyme phosphoenolpyruvate carboxykinase and downregulating insulin signalling in the muscle. In addition, it was suggested that RBP4 may inhibit the phosphorylation of the insulin receptor [49]. To date the results are still conflicting in humans as some studies confirmed this relationship [50,51,52,53] while others did not [49,54,55,56,57,58,59].

## 4. Molecular Mechanism for Retinoid Action in Adipose Tissue

The question that arises is what are the potential mechanisms of action by which retinoids and retinoid binding proteins affect adipose tissue biology and/or energy metabolism? Adipogenesis is governed by a complex regulatory cascade that has been extensively studied in committed clonal cell lines including 3T3-L1 cells (reviewed in [60,61]). In general terms, it involves the induction of members of the CCAAT/enhancer binding protein family (C/EBP) and PPARγ. Early in adipogenesis, C/EBPβ expression is induced, followed by the induction of PPARγ and C/EBPα expression. Both PPARγ and CEBPα lead to activation or enhanced expression of adipocyte markers [61]. PPARγ is considered the master regulator of adipogenesis and in its absence adipocyte differentiation cannot proceed [60]. Table 1 provides a summary of retinoid action described in the following sections. 

**Table 1:** Retinoid action in adipocyte differentiation (details see text).

Protein	Action in adipocyte differentiation
Retinol-binding protein (RBP/RBP4)	Increased expression in adipocytes is associated with increased systemic insulin resistance [48]
Retinaldehyde Dehydrogenase, type I (RALDH1)	Present in mature adipocytes it is involved in maintaining adipocyte differentiation. Lack of Raldh1 results in lack of adipocyte formation through modulating PPAR and RXR action [26]
Cellular-retinol binding protein, type I (CRBP-I)	Present in preadipocytes it is involved in inhibiting preadipocyte differentiation. Lack of CRBP-I leads to enhanced adipocyte differentiation and increased PPARγ activity [46]
Cellular-retinol binding protein, type III (CRBP-III)	Present in adipocytes supports differentiation of adipocytes. Lack of CRBP-III leads to decreased adipose tissue development and decreased PPARγ activity [47]
Cellular-retinoic acid binding protein, type II (CRABP-II)	Present in preadipocytes and is important in retinoic acid mediated inhibition of preadipocyte differentiation via RARs [62]
Beta-carotene monoxygenase (BCMO)	Present in adipocytes and it facilitates retinoic acid generation from β-carotene. Lack of BCMO activity leads to increased adipocyte differentiation and increased PPARγ expression [16]

### 4.1. Retinoic Acid, β-carotene and the Repression of Adipogenesis

It has long been appreciated that all-*trans*-retinoic acid can inhibit adipocyte differentiation by binding to and activating retinoic acid receptor (RAR) [63,64,65]. All-*trans*-retinoic acid inhibits adipogenesis early in the differentiation process by blocking the transcriptional activity of C/EBPβ [66]. Suppressed C/EBPβ activity lowers the expression of PPARγ, thus decreasing adipogenesis. In addition, retinoic acid may affect the cell cycle, keeping cells in a proliferative state rather than allowing growth arrest, which is essential for adipogenesis [67]. Retinoic acid no longer has an inhibitory effect on adipocyte differentiation if added to the cell culture after early differentiation is completed [16,68]. 

Retinoic acid applied to mature adipocytes appears to induce lipolysis and influence the production of several adipokines that are secreted into the tissue culture media. However, β-carotene and the subsequent intracellular generation of retinoic acid decreased adipocyte differentiation after early differentiation providing mechanistic data for the increased adiposity observed in mice lacking Bcmo1 [15]. The addition of β-carotene to 3T3-L1 cells during the mid stage of differentiation (day 5) results in a repression of adipocyte differentiation and loss of lipids [16]. Inhibition of the activity of Bcmo1 abolishes this effect. Interestingly, the gene for Bcmo1, which is a PPARγ target gene, is highly expressed in mature adipocytes, allowing for β-carotene to be converted to retinoic acid. The addition of β-carotene, however, leads to repression of PPARγ expression in the cell model, through a mechanism has not yet been identified. 

This differential effect of retinoic action on adipocyte differentiation, *i.e.*, that retinoic acid has an inhibitory role at early but not at later stages of the differentiation process may be explained by the fact that retinoic acid can be transported to the nucleus via two binding proteins, namely CRABP-II or FABP5 [38]. CRABP-II will deliver retinoic acid for binding to RAR while FABP5 will deliver retinoic acid to PPARβ/δ. Thus, in cells that express high levels of CRABP-II, retinoic acid will activate RAR and, conversely, in cells high in FABP5 will activate PPARβ/δ and their respective downstream pathways [38]. Recently, Berry *et al.* [62] provided data indicating that CRABP-II is highly expressed during early adipocyte differentiation but its expression is repressed during later stages. The lack of CRABP-II after early differentiation may thus explain that retinoic acid only inhibits differentiation early but not in later stages of differentiation. 

### 4.2. Effect of Retinaldehyde on Adipogenesis

Similar to retinoic acid, retinaldehyde is also capable of inhibiting adipogenesis *in vitro* and *in vivo*. As shown above (section 3.2.2), in the absence of Raldh1, increased intracellular retinaldehyde levels are accompanied by the development of decreased adiposity [26]. In contrast to the effect of retinoic acid on adipogenesis, the effect of retinaldehyde on adipogenesis might be due to the repression of PPARγ activity [26] pointing to retinaldehyde as a transcriptional regulator. Retinaldehyde appears to be able to bind both PPAR and RXR resulting in a repression of receptor activation and thus offering an explanation for retinaldehyde mediated inhibition of adipocyte differentiation. 

### 4.3. CRBP-I and CRBP-III Have Opposing Effects on Adipogenesis

We have recently shown that CRBPs are important participants in adipogenesis. Both CRBP-I and CRBP-III are expressed in adipose tissue with CRBP-I present in preadipocytes and CRBP-III in adipocytes. We have identified CRBP-III to be a PPARγ target gene and thus examined whether it participates in adipogenesis [47]. Using the 3T3-L1 cell model we showed that CRBP-III is expressed during mid- and late-stage differentiation [47]. To test the role of CBRP-III *in vivo* we used our CRBP-III knockout mouse model. When fed a high-fat diet, mice lacking CRBP-III developed decreased adiposity. Based on our *in vitro* data, it appears that lack of CRBP-III, PPARγ is markedly downregulated indicating that CRBP-III is important in maintaining adipogenesis (unpublished). In contrast, our data indicate that CRBP-I is an important inhibitor of adipogenesis [46]. Lack of CRBP-I *in vivo* led to increased adiposity. Similarly, *in vitro* silencing of CRBP-I led to enhanced adipocyte differentiation in 3T3-L1 preadipocyte cells and mouse embryonic fibroblasts (MEF). In addition, although CRBP-I is specifically expressed in preadipocytes it does not appear to regulate known inhibitory genes of adipogenesis. Our data point to a role of CRBP-I affecting PPARγ function. Further examination of molecular markers revealed that the expression and activity of PPARγ but not of other transcriptional regulators important during the different stages of differentiation were specifically increased in the absence of CRBP-I *in vitro* and *in vivo*. Interestingly, retinoid homeostasis was not altered in cells lacking CRBP-I. This finding is very similar to data presented on the enzyme retinol saturase (RetSat), where the function of RetSat in adipocytes was not readily explained by its enzymatic role of generating 13,14-dihydroretinol from retinol [69]. Taken together CRBP-I and CRBP-III appear to play important opposing roles in adipogenesis with CRBP-I repressing and CRBP-III maintaining adipogenesis.

## 5. Summary

In summary, retinoid metabolism is intricately involved in adipose tissue biology and thus affects whole body glucose and lipid homeostasis. Dietary studies and genetically engineered mouse models provide valuable insights into the roles of retinoid metabolism in adipose tissue biology. Several mouse models with disruption in different parts of retinoid metabolism provide evidence for a role of retinoid in adipose tissue biology and energy homeostasis. On a molecular level it has long been appreciated that retinoic acid can inhibit adipogenesis. However, in recent years new research has emerged pointing to new molecular pathways for retinoid functioning. Results from these studies suggest that alterations of retinoid metabolism affect the activity of the master regulator PPARγ [16,26,46,47,69]. The specific mechanisms remain to be studied. Ongoing studies will offer new insights into adipose physiology and the mechanisms for the regulation by retinoids of glucose and lipid metabolism. These studies will contribute to further understanding the factors important to adipose tissue development and may lead to future therapeutic interventions. These may include the development of small molecules that directly target adipose tissue development. 
